# Durable Clinical Benefit of Pertuzumab in a Young Patient with BRCA2 Mutation and HER2-Overexpressing Breast Cancer Involving the Brain

**DOI:** 10.1155/2016/5718104

**Published:** 2016-04-18

**Authors:** Anna Koumarianou, Christina Kontopoulou, Vassilis Kouloulias, Christina Tsionou

**Affiliations:** ^1^Hematology-Oncology Unit, Fourth Department of Internal Medicine, National and Kapodistrian University of Athens, Attikon University Hospital, Rimini 1, Haidari, 12462 Athens, Greece; ^2^Second Radiology Department, Attikon University Hospital, National and Kapodistrian University of Athens, Rimini 1, Haidari, 12462 Athens, Greece; ^3^Radiotherapy Unit, Second Radiology Department, Attikon University Hospital, National and Kapodistrian University of Athens, Rimini 1, Haidari, 12462 Athens, Greece; ^4^Maternity-Health, 30 Papanikoli Street, Halandri, 15232 Athens, Greece

## Abstract

Patients with HER2-positive breast cancer and brain metastases have limited treatment options, and, as a result of their poor performance status and worse prognosis, they are underrepresented in clinical trials. Not surprisingly, these patients may not be fit enough to receive any active treatment and are offered supportive therapy. BRCA2 mutations are reported to be rarely associated with HER2-overexpressing advanced breast cancer and even more rarely with brain metastases at diagnosis. We report on a BRCA2-positive breast cancer patient with metastatic disease in multiple sites, including the brain, and poor performance status who exhibited an extraordinary clinical and imaging response to the novel anti-HER2 therapy pertuzumab after multiple lines of therapy including anti-HER2 targeting. To our knowledge, the clinicopathologic and therapeutic characteristics of this patient point to a unique case and an urgent need for further investigation of pertuzumab in patients with brain metastases.

## 1. Introduction

Brain involvement of metastatic breast cancer is largely dependent on the molecular subtype of the disease, and in the case of HER2-overexpressing tumors, it affects 30% of patients and is associated with devastating symptoms and quality of life [1, 2].

Several studies confirm that the median survival from diagnosis of brain metastases is from 6 months for patients who do not receive trastuzumab to 13 months for those who receive trastuzumab [1, 3, 4]. With few exceptions, there are hardly any prospective phases II-III clinical studies interrogating the role of anti-HER2 drugs in brain involvement [5, 6]. A plausible explanation for such a serious underrepresentation of patients in clinical trials is the poor performance status of this patient subgroup due to the tumoral involvement of the central nervous system. A recent study combining trastuzumab and pertuzumab for patients with metastatic disease resulted in survival improvement of 15.7 months [7]. Because this study did not include patients with brain metastases, there is no established experience about such anti-HER2 therapy's potential administration and benefit in patients relapsing after trastuzumab and lapatinib. With regard to patients with HER2-overexpressing metastatic breast cancer who are BRCA2 carriers, there is a scarcity of information in the published literature [8], so there is not much data on their survival and responses to the available treatments. However, a recent meta-analysis including breast cancer patients who are BRCA2 mutation carriers indicated no survival differences compared to patients without BRCA2 mutations [9].

## 2. Case Presentation

A 34-year-old female with abdominal pain and headache presented at the emergency department in November 2009. Upon clinical examination, she had a red, hard, fixed right breast with a fixed lymph nodal mass in the right axilla. She was living in the outskirts of Sparta with her husband and two children. She was never a smoker or an alcohol drinker.

Her past medical history included a pituitary adenoma; she had no brothers or sisters, but she had a remarkable family history, as her father and uncle died of metastatic breast cancer.

Her imaging with computed tomography (CT) and laboratory investigations revealed a single brain and multiple lung, liver, and bone metastases. A full blood count and biochemistry were normal except for the following: hemoglobin: 9.8 g/dL (normal value; nv 12–16); aspartate aminotransferase: 78 U/L (nv 5–40); alanine aminotransferase: 96 U/L (nv 5–35); gamma-glutamyl transferase: 216 U/L (nv 7–49); alkaline phosphatase: 261 U/L (nv 25–125); lactic acid dehydrogenase: 778 U/L (<250); and tumor markers: Ca 15-3 >1000 U/mL (nv < 31) and CEA > 50 ng/mL (nv < 5).

Histologic and immunohistochemical examination of a true-cut biopsy revealed an invasive ductal adenocarcinoma, grade III differentiation, estrogen receptor (ER) strong nuclear positivity in 70% of cells, progesterone receptor (PR) nuclear positivity in less than 3%, HER2 strongly positive (+3), Ki-67 nuclear positivity in 40% of cells, and p53 nuclear positivity in 60% of cells according to the histopathology.

**Figure 1:**
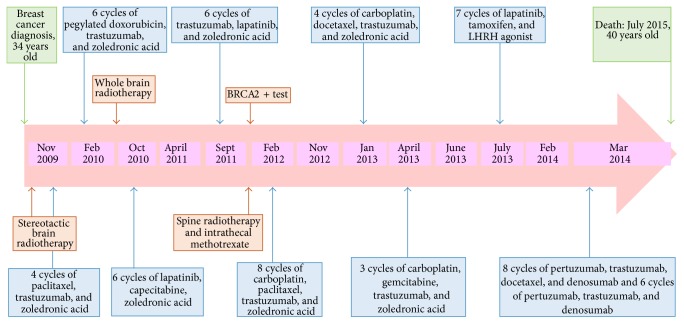
Timeline of patient's diagnosis and treatments.

Following stereotactic brain radiotherapy to the single lesion, the patient was treated according to the 2009 NCCN first-line strategy recommendations, which included paclitaxel, trastuzumab, and zoledronic acid [10]. Reevaluation after four cycles revealed disease progression (PD) in the lung and liver according to the RECIST criteria of response. A second line of therapy was initiated and included pegylated doxorubicin, trastuzumab, and zoledronic acid [11]. The choice of this combination was primarily based on patient's desire to avoid further alopecia. The first reevaluation at 3 months indicated partial response (PR) at all sites, including the breast, but a second assessment at 6 months revealed a relapse in the brain and lung. Following whole brain radiation, the patient was treated with a third-line therapy consisting of combination therapy with lapatinib, capecitabine, and zoledronic acid [12]. At CT reevaluation at 6 months, there was leptomeningeal dissemination and a vertebral thoracic mass (T12). After cytologic confirmation of leptomeningeal disease by lumbar puncture, the patient was treated with three courses of intrathecal methotrexate and radiotherapy to the spine (T12 level). The reevaluation showed a negative cerebrospinal fluid cytology but PD at all other sites: brain, lung, and liver. The patient received three further lines of combination chemotherapy including carboplatin, following a positive BRCA2 test [13–16], experiencing brief responses as described in Figure 1.

**Figure 2:**
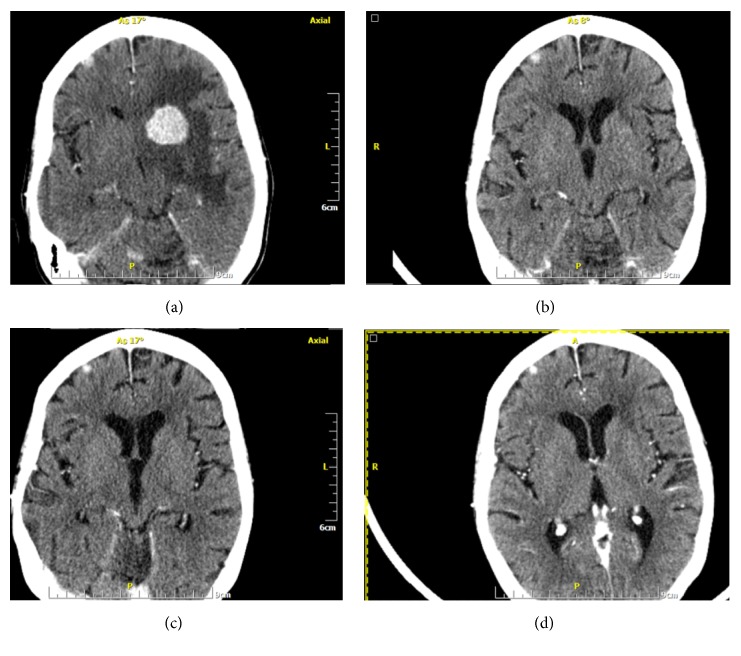
Brain computed tomography at baseline (a) and after 3 (b), 9 (c), and 12 (d) months of treatment with pertuzumab, trastuzumab, and taxane-based chemotherapy.

Following PD after the seventh line of therapy based on the combination of carboplatin, gemcitabine, and trastuzumab [16], the patient was physically and psychologically exhausted and willing to proceed with some form of active treatment. Thus, an eighth line of orally administered therapy at home was initiated and included lapatinib and hormonal therapy. Seven months later, the patient deteriorated clinically (PS ECOG/WHO: 3-4) and arrived at the outpatient department in a wheelchair. Total body CT scans revealed PD in all sites, including the brain. Based on new NCCN guidelines and following discussion with the patient and her family, a ninth line of therapy was initiated, including the novel combination of pertuzumab/trastuzumab, docetaxel, and denosumab [17]. The patient's performance status gradually improved with CT reevaluation at 4 months, revealing a PR at all sites, including the brain (Figure 2). The clinical and imaging response continued to improve until 12 months later when a “plateau” was achieved. The patient continued on maintenance therapy with pertuzumab/trastuzumab/denosumab without chemotherapy for further six months. During all this time, she was walking and active at home taking care of her children, but when this time elapsed the patient rapidly deteriorated and succumbed to her disease.

## 3. Discussion

We report on a young patient who is a BRCA2 mutation carrier diagnosed with HER2-positive breast cancer with visceral and brain metastases. Having received nine lines of anti-HER2 treatment, including a platinum agent, and radiotherapy to the brain, there were only two available options: referral to the supportive care team or a tenth line of treatment with the recently approved pertuzumab. The CLEOPATRA trial had indicated an excellent survival benefit for patients with HER2-positive metastatic disease treated with pertuzumab but provided no data on patients with brain metastases, as these were excluded [7]. At that time, the EMILIA trial on TMD-1 had just been completed, and the treatment was shown to result in very good response rates [18], but it was not yet locally approved or readily available for the patient. A recently reported phase II trial on pertuzumab/trastuzumab combination without chemotherapy in patients who had progressed under trastuzumab based regimen indicated a 50% clinical benefit rate and a 5.5-month median progression-free survival [19]. Our patient received one cycle of pertuzumab, trastuzumab, and docetaxel and experienced rapid symptomatic relief, indicating the need for continuation.

Patients with HER2-positive breast cancer are living longer due to effective treatment, and as a result, brain metastases are now most common [3]. Patients like ours are usually not involved in clinical trials due to their poor performance status that is allegedly related to bad prognosis, and the optimal therapy remains an open question. Until today, only one case with brain metastases has been reported to experience complete response under second-line combination of pertuzumab-trastuzumab and docetaxel [20]. A retrospective study including patients rechallenged with trastuzumab after second-line lapatinib therapy for CNS involvement indicated a rather poor median OS of 17.3 (3.4–32.2) months [21]. Although monoclonal antibody penetration in the CNS has been questioned [22, 23], recently, a TDM-1 study [24] and a PET imaging analysis employing a radiolabeled trastuzumab-DOTA conjugate [25] have indicated indirectly and directly that monoclonal antibody targeting of brain metastases occurs effectively.

There are only a few studies available evaluating the role of anti-HER2 treatments in patients with disease metastasized to the brain, and these include small numbers of patients. Among these is one retrospective exploratory analysis of the phase III EMILIA study including 92 patients, of whom 43 were treated with T-DM1 and had an overall survival time of 26.8 months, compared to 12.9 months in the lapatinib/capecitabine group (49 patients) [24]. Additional studies include a multicenter phase II study including 242 patients treated with lapatinib [6], one phase III study of 36 patients with brain metastases treated with trastuzumab and lapatinib [5], one retrospective analysis of 432 patients, indicating that patients treated with both trastuzumab and lapatinib after developing metastases had significantly longer survival than patients treated with either agent alone [26], and one retrospective study including 79 patients treated with trastuzumab [27]. Based on the clinical activity observed in the EGF104900, LANDSCAPE, and EMILIA studies, the most active treatment options for HER2-positive breast cancer patients with brain metastases include trastuzumab/lapatinib [28], capecitabine/lapatinib [29], and TDM-1 [24].

A major challenge faced in patients with metastatic disease, including those with breast cancer, is whether metastases develop according to a linear or a metastatic cascade model, which could lead to a separate molecular profile with important therapeutic implications [30].

## 4. Conclusions

Brain metastases are increasingly being acknowledged as the next major step to prolong survival in HER2-positive breast cancer. Our patient, combining systemic and local therapies, lived 67 months from the diagnosis of brain disease. The effectiveness observed in our patient indicated that the combination of pertuzumab/trastuzumab deserves further investigation in the context of larger studies. Nevertheless, it is necessary to assess the optimal anti-HER2 drug sequence in conjunction with efforts to redefine the role of radiotherapy and surgery in breast cancer patients with metastatic disease in the brain.
